# Native T1 mapping for assessment of the perilesional zone in metastases and benign lesions of the liver

**DOI:** 10.1038/s41598-020-69819-w

**Published:** 2020-07-30

**Authors:** Ute Lina Fahlenkamp, Katharina Ziegeler, Lisa Christine Adams, Sarah Maria Böker, Günther Engel, Marcus Richard Makowski

**Affiliations:** 1 Department of Radiology, Charité Universitätsmedizin Berlin, Charitéplatz 1, 10117 Berlin, Germany; 2 Department of Radiology, Klinikum rechts der Isar der TU München, Ismaninger Straße 22, 81675 Munich, Germany

**Keywords:** Biomarkers, Gastroenterology, Molecular medicine

## Abstract

Adjacent to hepatic metastases, liver parenchyma is often histopathologically altered even if its visual appearance on native magnetic resonance (MR) images is blunt. Yet, relaxation properties in MR imaging may show structural changes prior to visual alteration, and therefore, the aim of this study was to investigate whether T1 relaxation times in the perilesional zone differ between metastases and benign lesions. A total of 113 patients referred for MRI were included prospectively. Images were assessed for metastases, solid benign lesions and cysts, and regions-of-interest were drawn on T1 maps including the focal lesion and a close (inner perilesional zone = IPZ) and a larger perilesional zone (outer perilesional zone = OPZ). Simple ratios between these zones, as well as a gradient ratio between the IPZ and the entire perilesional zone (EPZ) were calculated. Within the collective, 44 patients had lesions of one or two entities. For metastases, the simple ratio between IPZ and OPZ as well as the mean EPZ gradient was significantly higher than for both solid benign lesions and cysts. Lesion size was not a significant covariate. We conclude, that native T1 properties of the perilesional zones differ significantly between malignant and both solid and cystic benign lesions.

## Introduction

It is well known that liver parenchyma adjacent to hepatic metastases is often altered due to edema, vascular or bile duct proliferations^1,2^, as well as due to malignant infiltration in some cases. Whereas appearance on native images is often blunt, an imaging correlate for this can sometimes be seen as a so-called rim enhancement^3^. Due to the benign nature, perilesional alterations should not be as pronounced around non-malignant hepatic lesions, and especially around cysts, which should—apart from a possibly compressing effect in very large types—not have any effect on perilesional relaxation times. Therefore, to quantify differences in the perilesional zone could be of value for confident diagnostic decision making.

In general, a native approach to quantify the perilesional zone would be preferable to a contrast-enhanced approach as recent research^4–6^ as well as special contraindications in selected patients make a reduced use of MR contrast agents desirable but also as the contrast-enhanced technique can be influenced by other aspects, such as size and vascularization of the lesion^7^, or by the type and amount of the contrast agent. Whereas vascular proliferation or sinusoidal congestion are probably best to be detected under appliance of contrast agents, most of the alterations in the perilesional zone found in histopathology, i.e. edema and fibrosis, can also be detected by native imaging techniques.

For tissue characterisation in cardiac imaging, native T1 mapping has shown its value allowing for precise quantification at rather short imaging times^8,9^. In liver imaging, mapping techniques have mainly been evaluated using the hepatocyte specific contrast agent gadoxetate disodium^10–12^ whereas native T1 mapping techniques are not yet well established yet.

Therefore, the study was set up to evaluate whether the on native imaging visually inconspicuous liver parenchyma adjacent to a focal lesion shows altered T1 relaxation times compared to the more peripherally located liver parenchyma and, if so, whether there is a difference in terms of this gradient between metastases and solid benign lesions as well as cysts.

## Material and methods

### Study population

The study was prospectively approved by and registered with the local ethics committee (Ethikkommission der Charité, Ethikausschuss I am Campus Charité—Mitte, EA1/334/16) and the methods were carried out in accordance with the relevant guidelines and regulations. Written informed consent was obtained from all participants.

From December 2016 to February 2020, patients referred for an MR examination of the liver with gadoxetate disodium were screened for eligibility. Inclusion criteria were thus exclusion or assessment of metastases in patients with extrahepatic tumours, exclusion or assessment of hepatocellular carcinoma in patients with cirrhosis, tumour assessment in patients with suspected cholangiocellular carcinoma, suspected biliary disease, and hemochromatosis. Exclusion criteria were age younger than 18 years, pregnancy, metallic implants or functional devices not eligible for MR examination, claustrophobia, a history of allergic reaction to gadoxetate disodium, and a glomerular filtration rate below 30 ml/min.

All in all, 113 patients (57 male and 56 female; age range 19–87 years; mean 56.8, SD 15.0 years) were included in the study. Within these, indications for referral were exclusion or assessment of metastases in patients with extrahepatic tumours (n = 74), exclusion or assessment of hepatocellular carcinoma in patients with cirrhosis (n = 20), tumour assessment in patients with suspected cholangiocellular carcinoma (n = 6), primary sclerosing cholangitis (n = 3), cholestasis (n = 2), known focal nodular hyperplasie or adenoma for follow-up (n = 3), hemochromatosis (n = 2), and focal lesions of unknown entity but without extrahepatic tumour, cirrhosis or suspicion for cholangiocellular carcinoma (n = 3).

### Imaging protocol

Images were acquired on a clinical 1.5 T MR scanner (Avanto; Siemens Healthineers, Erlangen, Germany) with a 16-channel body-phased-array coil. All included patients underwent standard liver MR imaging using the hepatocyte specific contrast agent gadoxetate disodium (Gd-EOB-DTPA, Primovist) which includes an axial T1-weighted spin echo sequence, an axial fat-saturated T2-weighted turbo spin echo sequence acquired with a 2D navigator for abdominal imaging (2D Prospective Acquisition CorrEction, PACE), an axial T1-weighted dual echo sequence, and axial T1 GRE (gradient recalled echo) sequences for dynamic imaging before and 15, 55 s and 2, 5, 10 and 20 min after contrast agent administration and a coronally orientated T1 GRE sequence for the hepatobiliary phase at least 20 min after contrast agent administration^13^.

#### Study sequences

Apart from the clinical routine image protocol, patients received native steady-state precession readout single-shot Modified Look-Locker inversion recovery (MOLLI) sequences in the axial plane.

T1 maps were calculated automatically on a pixel-by-pixel basis, and displayed on a 12-bit lookup table with a visible color-coded map, on which the signal intensity of each of the pixels reflects their absolute T1 value.

The imaging parameters for the study sequence are shown in Table 1.

**Table 1:** Imaging parameters of the study sequence. *MOLLI* modified Look-Locker Inversion Recovery *TR* repetition time *TE* echo time *FoV* field of view *TI* inversion time.

Sequence	MOLLI
Scan plane	Axial
Voxel size (mm^3^)	2.4 × 1.6 × 6.0
Number of slices	3
Slice thickness (mm)	6
TR/TE (ms)	912/1.08
Averages	1
FoV (mm)	320
Flip angle (°)	35
Bandwidth (Hz/Px)	1,028
Fat saturation	None
Parameter map type	T1 map
Number of inversions	3
MOLLI TI start (ms)	90
MOLLI TI increment (ms)	80
MOLLI trigger delay (ms)	160

### Image analysis

All imaging sequences were analysed on standard workstations (Centricity PACS, Radiology RA1000, General Electrics).

Region-of-interest (ROI) placement was done manually by a reader blinded to the diagnosis of the focal lesion according to the following approach: first, a ROI entirely encircling the focal lesion (or cyst) was placed on the T1 map, adding—if possible—a circumferent narrow safety distance of 1–3 mm. Then, in a circumferent distance in the adjacent liver parenchyma of 5 mm, a larger ROI (zone 1) was added, entirely encircling the first ROI under avoidance of larger vessels, other focal lesions, and extrahepatic structures. The third and largest ROI (zone 2) was drawn including the two other ones under adherence to the same requirements. T1 relaxation times as well as the area of the ROIs (in mm^2^) were recorded.

**Figure 1:**
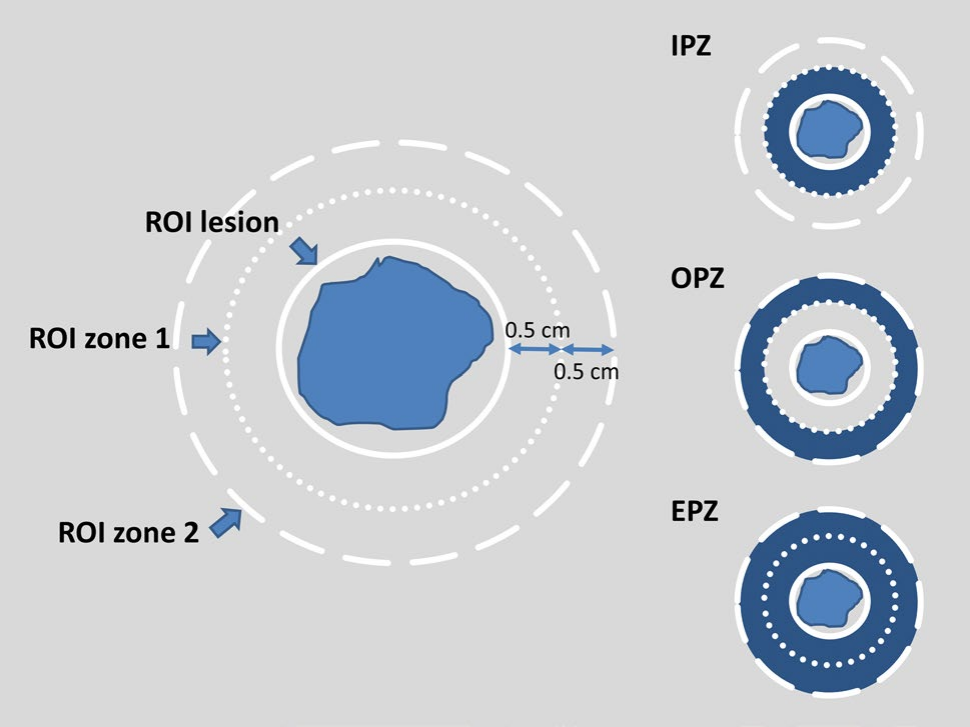
Sketch illustrating ROI placement as well as definitions of the inner peripheral zone (IPZ), the outer peripheral zone (OPZ) and the entire peripheral zone (EPZ).

**Figure 2:**
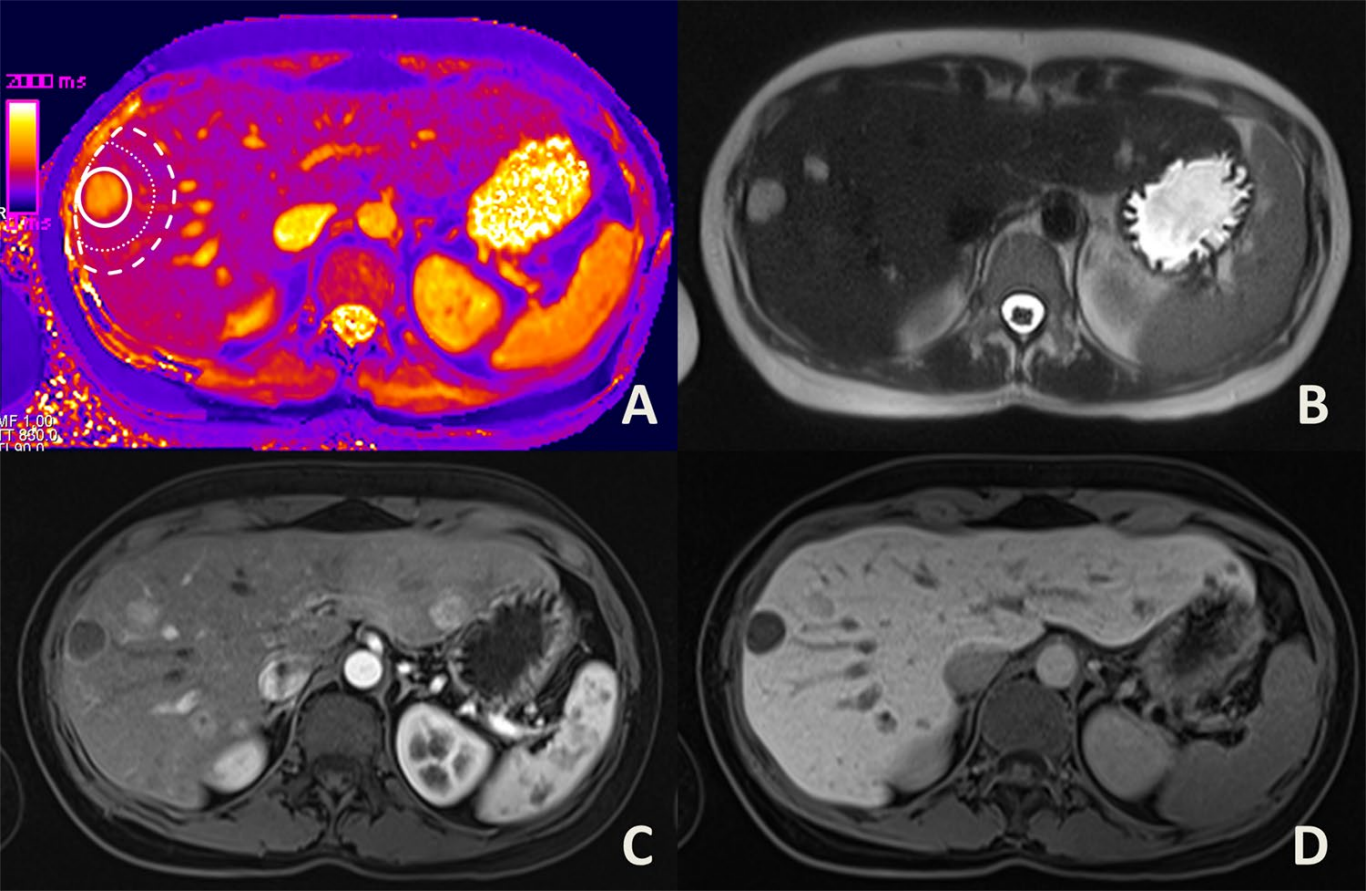
Placement of a region of interest (**A**) around a metastasis in a 33 year-old female patient with squamous cell carcinoma of the nasal sinus. As the lesion is located subcapsular, the peripheral zones (dotted and dashed circles) are placed excentrically around the lesion ROI (continuous circle), under careful avoidance of larger vessels, other focal lesions or extrahepatic structures. Additionally, a T2-weighted (**B**), an arterial phase (**C**), and the hepatobiliary phase (**D**) images are given.

**Figure 3:**
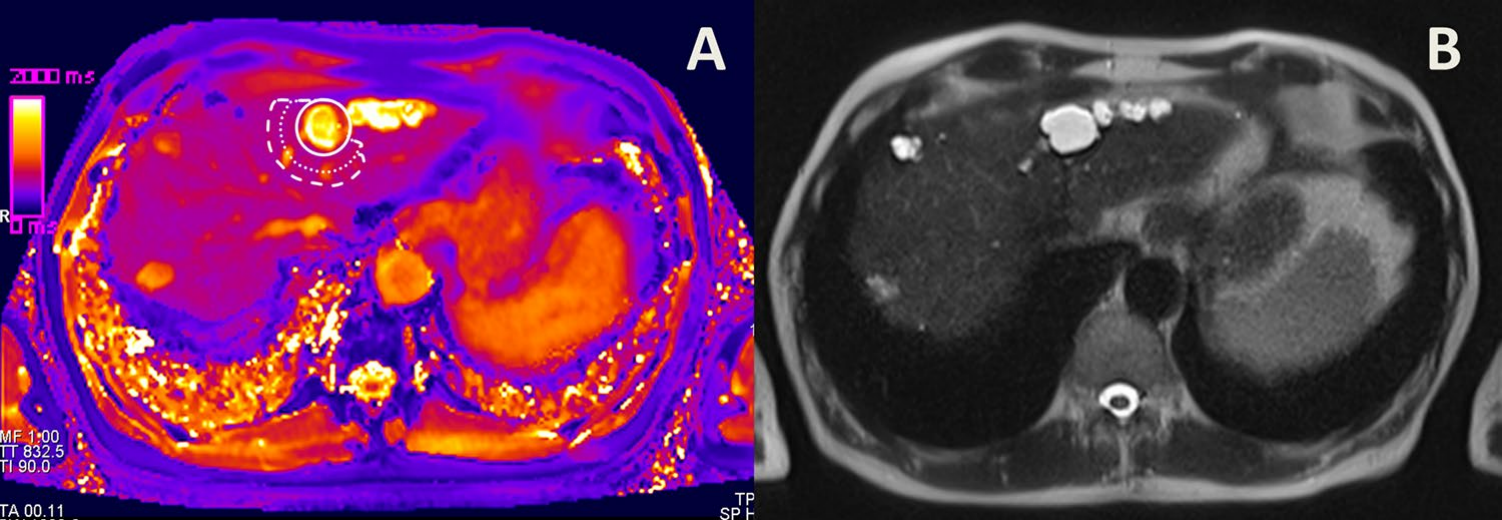
Placement of a region of interest (**A**) around a cyst in a 65 year-old male patient with renal cell cancer. (**B**) displays the native T2 weighted image.

**Figure 4:**
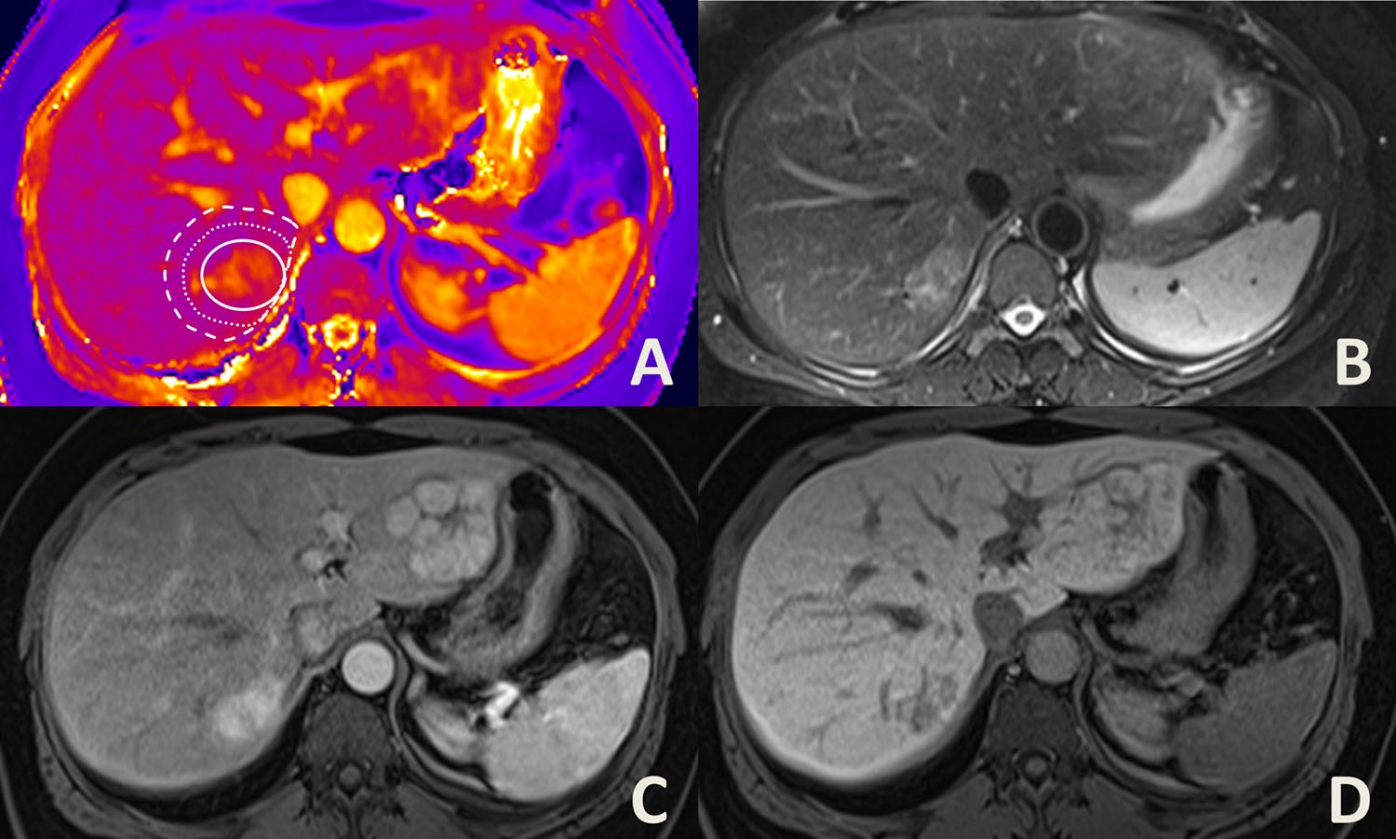
Placement of a region of interest (**A**) in a 48-year old female patient with multiple focal nocular hyperplasias. (**B**) Displays a T2 fat-saturated image, whereas (**C**) shows the portal venous filling phase, and (**D**) the hepatobiliary phase after injection of gadoxetate disodium.

A sketch of the approach is given in Fig. 1. Additionally, Figs. 2, 3, 4 show examples of ROI placement in a patient with metastases, in a patient with cysts, and in a patient with benign liver lesions.

Relaxation times of the inner perilesional zone (IPZ), as well as of the outer perilesional zone (OPZ) were calculated according to the following formula.

$$IPZ = \frac{area1}{{area1 - area \;lesion}}*(ROI\;1 - \left( {area\; lesion/area1*ROI\; lesion} \right))$$

$$OPZ = \frac{area2}{{area2 - area1}}*(ROI2 - \left( {area1/area2*ROI1} \right))$$

Additionally, for comparison, to minimize the impact of possible partial volume effects in the inner zone caused by the lesion, the relaxation time of the entire peripheral zone, including the inner perilesional zone was calculated.

$$EPZ = \frac{area2}{{area2 - area \;lesion}}*(ROI2 - \left( {area\;lesion/area2*ROI\; lesion} \right))$$

The simple ratio (R1) between the IPZ and the OPZ was calculated as follows:

$$R1 = IPZ/OPZ$$

The EPZ gradient (R2), as a measure for the relation between the inner perilesional zone (IPZ) and the entire perilesional zone (EPZ), was calculated as follows:

$$R2 = 100*\left( {IPZ - EPZ} \right)/EPZ$$

In order to test the robustness of the measurement technique, a random sample of 10% of test patients (n = 12 lesions in 12 different patients) was repeated by the same reader after an appropriate time interval.

### Statistical analysis

All statistical analyses were performed using SPSS Version 25 (IBM Corporation, New York, USA). Means and standard deviation of T1 relaxation times as well as areas of measured ROIs and secondary values discussed above were calculated for metastases, benign lesions and cysts. Comparisons of the three lesion types were performed with generalised estimating equations (GEE) using the identity link function with robust variance estimate. Since the number of lesions considered for each patient ranged from 1 to 7, data are not independent. GEE models account for within-subject correlations arising when outcomes are measured on the same patient more than once^14^. For each outcome, the three group comparisons with metastases vs. benign lesions, metastases vs. cysts and benign lesions vs. cysts were performed. Lesion type was defined as a factor and lesion area was defined as a covariate. In order to avoid inflation of the α-level due to multiple testing, the global null hypothesis was tested, that all three lesion types have the same mean first, and the three group comparisons were only considered, if the global test was significant. The two-sided level of significance was α = 0.05. Intra-reader reliability was tested using intra-class correlation coefficients ICC^15^. Receiver operating curve (ROC) analyses were performed to determine the capacity to distinguish between malign and benign (both solid and cystic) lesions.

## Results

All in all, 44 patients within the collective had focal liver lesions of one or two entities. 28 patients showed only metastases on the T1 maps, 2 patients had metastases and cysts, 5 patients had benign lesions, one had a benign lesion and a cyst, and 8 patients had only cysts.

The hepatic metastases displayed on the T1 maps in 30 patients were due to cholangiocellular carcinoma (n = 2), neuroendocrine carcinoma (n = 8), colonic cancer (n = 6), breast cancer (n = 5), squamous cell carcinoma (n = 2), angiosarcoma (n = 1), melanoma (n = 1), extragonadal tumour (n = 1), renal cell carcinoma (n = 1), and pancreatic tumour (n = 3). Thereof, several patients showed numerous lesions, leading to an overall number of 83 measureable metastases. 6 patients had one or several hepatic cysts, which led to 26 cysts displayed on the T1 maps. Benign solid hepatic lesions were present in 5 patients, which led to 17 lesions displayed on the T1 maps, as several patients had multiple lesions. These were hemangioma (n = 4), adenoma (n = 5), and focal nodular hyperplasia (n = 8).

### Metastases

Mean relaxation time of the metastases was 947.8 ms (565 to 1651 ms, SD 204.1). The IPZ adjacent to metastases had a mean relaxation time of 628.1 ms (SD 92.0), and the OPZ of 588.1 ms (SD 77.9 ms). The EPZ had a mean relaxation time of 604.4 ms (SD 81.6).

The means size of the ROI of the lesion was 1,035.3 mm^2^ (44–7,566 mm^2^, SD 1523.6), of zone 1 1736.2 mm^2^ (283–8,899 mm^2^, SD 1736.2), and of zone 2 2,688.7 mm^2^ (610–9,829 mm^2^, SD 2,135.7). Thus, the area of the IPZ was 700.9 mm^2^, and of the OPZ 952.5 mm^2^. The EPZ measured 1653.4 mm^2^, accordingly.

### Cysts

Mean relaxation time of the cysts was 1,298.2 ms with a standard deviation of 304.4 ms. The IPZ adjacent to cysts had a mean relaxation time of 617.9 ms (SD 56.6 ms), and the OPZ of 606.4 ms (SD 56.3 ms). The EPZ had a mean relaxation time of 612.8 ms (SD 53.5 ms).

The size of the ROI of the cysts was 1,151.3 mm^2^ (32–71,770 mm^2^, SD 2,045.6), of zone 1 1846.6 mm^2^ (166–8,860 mm^2^, SD 2,632.4), and of zone 2 2,583.9 mm^2^ (288–10,840 mm^2^, SD 3,162.9). Thus, the area of the IPZ was 695.3 mm^2^, and of the OPZ 737.3 mm^2^. The EPZ measured 1,432.6 mm^2^, accordingly.

### Benign lesions

Mean relaxation time of the benign lesions was 791.4 ms with a standard deviation of 137.8 ms. The IPZ adjacent to the benign lesion had a mean relaxation time of 658.1 ms (SD 100.4), and the OPZ of 641.8 ms (SD 97.4). The EPZ had a mean relaxation time of 653.4 ms (SD 103.4 ms).

The size of the ROI of the benign lesions was 489.5 mm^2^ (181–1,567 mm^2^, SD 350.9), of zone 1 906.6 mm^2^ (415–2,036 mm^2^, SD 468.7), and of zone 2 1,389.2 mm^2^ (637–2,705 mm^2^, SD 538.0). Thus, the area of the IPZ was 417.1 mm^2^, and of the OPZ 482.6 mm^2^. The EPZ measured 899.7 mm^2^, accordingly.

### Comparison

For metastases, the simple ratio between the inner and the outer peripheral zone was 1.07 (0.98–1.37, SD 0.07). For benign lesions the simple ratio was 1.03 (0.97–1.18, SD 0.05) and for cysts, it was 1.02 (0.92–1.09, SD 0.05). The difference was statistically significant for metastases vs. benign lesions (p = 0.012) and metastases vs. cysts (p = 0.001) but not for benign lesions vs. cysts (p = 0.606). For all comparisons, lesion size was not a statistically significant covariate (p > 0.05).

The EPZ gradient, as a measure for the relation between the inner and the entire perilesional zone was 3.8 for metastases (− 1.4 to 23.0, SD 4.1). For cysts, the EPZ gradient was 0.8 (− 5.4 to 5.1, SD 2.8), and for benign lesions, it was 0.9 (− 7.1 to 5.8, SD 3.3). Again, the difference was statistically significant for metastases vs. benign lesion (p = 0.001) and cysts (p < 0.001) but not for benign lesions vs. cysts (p = 0.887). Lesion size was not a significant cofactor in any of the comparisons.

ROC analyses yielded an area under the curve (AUC) of 0.702 (95% CI 0.608–0.797; p < 0.001) for the EPZ gradient and an AUC of 0.672 (95% CI 0.576–0.769; p = 0.002) for the simple ratio.

Intra-reader reliability as a measure of robustness of the measurement technique was excellent with a mean ICC of 0.976 (range 0.952–0.998, p < 0.001).

**Figure 5:**
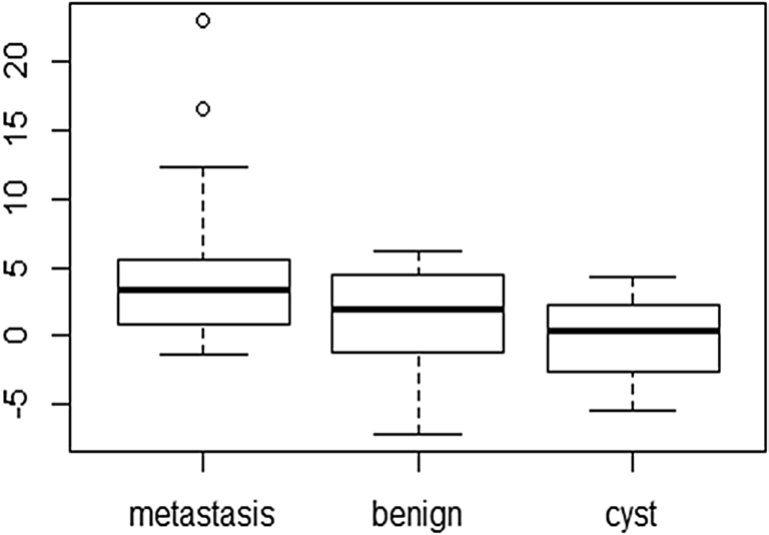
Box-and-whisker plot depicting the IPZ/EPZ gradient. The whiskers indicate variability outside the upper and lower quartiles, the individual points demonstrate outliers.

Mean relaxation times and ratios are given in Table 2. Additionally, a graphical representation of the findings regarding the EPZ gradient is given as Fig. 5.

**Table 2:** Mean relaxation times of the different focal liver lesions and their calculated ratios.

Relaxation time (mean)	Metastases	Benign lesion	Cysts
Lesion (ms)	947.8	791.4	1,298.2
IPZ (ms)	628.1	658.1	617.9
OPZ (ms)	588.1	641.8	606.4
EPZ (ms)	604.4	653.4	612.8
Simple ratio	1.07	1.03	1.02
EPZ gradient	3.8	0.9	0.8

## Discussion

In the present study we evaluated whether visually blunt liver parenchyma adjacent to a focal lesion shows altered T1 relaxation times compared to the more peripherally located liver parenchyma, and, if so, whether this gradient differs between metastases, benign solid lesions and simple cysts, which we estimated to be acceptable as a negative control. Our results show that there is a gradient in the parenchyma surrounding focal lesions with higher T1 values directly adjacent and lower T1 values more peripherally, and that this gradient is significantly higher adjacent to metastases compared to benign solid lesions.

Characterisation of different compositions of parenchyma by T1 and T2 mapping techniques is well established in cardiac imaging^16^. Whereas T2 relaxation time is mainly influenced by water, and thereby primarily reflects edema, T1 relaxation time is said to reflect changes in water content as well as in the local molecular environment^17^. In acute myocarditis, T1 mapping has even been found to be superior to T2 weighted CMR in detecting myocardial edema in the context of acute myocarditis^8^, even though other studies admit, that there is still no clear advantage of one mapping sequence over the other^18^.

Until now, the peritumoral zone in the liver has primarily been assessed by contrast-enhanced approaches, as peripheral rim enhancement of lesions on early phase post-contrast images was described as a characteristic finding of metastatic lesions^2,7,19,20^. As an underlying etiology malignant or inflammatory cell infiltration, desmoplastic reaction, surrounding parenchymal compression, and vascular as well as bile duct proliferation were discussed, which could be proven with histologic specimens in the experimental setting^19^, as well as in patients^2,20^. Apart from primarily vascular alterations leading to the hyperintense rim, i.e. peritumoral congestion, probably due to a bloodpool effect^2^, the majority of the other cellular and pericellular alterations named above and described histologically, probably also lead to alterations in native relaxation time.

In either way, the most obvious reason for the more prominently altered T1 relaxation time around metastases could be, that usually malignant lesions cause a surrounding edema, either by congestion, or inflammation^2,19^. Additionally, as T1 relaxation time is directly related to the diffusion coefficient of protons^21^, another reason for a gradient in T1 relaxation time surrounding focal lesions might be the mass effect, going along with flattening of parenchyma, which would also explain the slight gradient around benign lesions including cysts in some cases, although lesion size was not a significant covariate in the analyses we performed.

Approaches to classify focal lesions without contrast-agent are desirable not only due to the recent publications on safety issues concerning MR contrast agents^5,6,22^. But also for well-known risks including allergic reactions. The approach presented here might be a first step towards a new methodology, especially as more sophisticated computational approaches such as texture analysis show initial positive results for textural changes before their visual appearance, irrespective of the question whether the textural changes are a result of the metastatic cells themselves or rather a reflection of reactive changes in the surrounding liver parenchyma^23^.

## Limitations

Despite careful planning and execution, there are some limitations to our study that need to be discussed. Firstly, we had a rather small number of cysts and benign lesions in our study cohort, as patients only underwent exams for exclusion and assessment of malignant lesions, meaning that all benign lesions included represent mere incidental findings. Therefore, the study sample size is not large enough to provide a definite cut-off value for differentiation between benign and metastatic lesions, which also means that descriptors of diagnostic accuracy, such as sensitivity and specificity, could not be calculated. Another limitation is that histology cannot be given as a gold-standard: even though in most patients with metastases, hepatic metastatic disease had been confirmed by biopsy, it might possibly not be the lesion imaged that had been biopsied, and, in other cases, depending on the individual situation of the patient, diagnosis of hepatic metastatic disease was made on imaging features on MR images, and/or CT and/or contrast-enhanced ultrasound. The same goes for the benign lesions, which were not histologically proven, but usually correlated with ultrasound and/or CT.

## Conclusion

Native T1 mapping reveals perifocal alterations of T1 relaxation time around hepatic metastases which differ significantly from those around solid and cystic benign lesions. These findings may be developed further into an imaging marker of malignant lesions non-dependent on intravenous contrast administration.
